# Remote Patient Monitoring via Non-Invasive Digital Technologies: A Systematic Review

**DOI:** 10.1089/tmj.2016.0051

**Published:** 2017-01-01

**Authors:** Ashok Vegesna, Melody Tran, Michele Angelaccio, Steve Arcona

**Affiliations:** ^1^ Jefferson College of Population Health, Philadelphia, Pennsylvania.; ^2^ Novartis Pharmaceuticals Corporation, East Hanover, New Jersey.; ^3^ Scott & White Health Plan, Temple, Texas.

**Keywords:** *e-health*, *m-health*, *telehealth*, *telemedicine*

## Abstract

***Background:*** *We conducted a systematic literature review to identify key trends associated with remote patient monitoring (RPM) via noninvasive digital technologies over the last decade.* ***Materials and Methods:*** *A search was conducted in EMBASE and Ovid MEDLINE. Citations were screened for relevance against predefined selection criteria based on the PICOTS (Population, Intervention, Comparator, Outcomes, Timeframe, and Study Design)* *format. We included studies published between January 1, 2005 and September 15, 2015 that used RPM via noninvasive digital technology (smartphones/personal digital assistants [PDAs], wearables, biosensors, computerized systems, or multiple components of the formerly mentioned) in evaluating health outcomes compared to standard of care or another technology. Studies were quality appraised according to Critical Appraisal Skills Programme.* ***Results:*** *Of 347 articles identified, 62 met the selection criteria. Most studies were randomized control trials with older adult populations, small sample sizes, and limited follow-up. There was a trend toward multicomponent interventions (*n* = 26), followed by smartphones/PDAs (*n* = 12), wearables (*n* = 11), biosensor devices (*n* = 7), and computerized systems (*n* = 6). Another key trend was the monitoring of chronic conditions, including respiratory (23%), weight management (17%), metabolic (18%), and cardiovascular diseases (16%). Although substantial diversity in health-related outcomes was noted, studies predominantly reported positive findings.* ***Conclusions:*** *This review will help decision makers develop a better understanding of the current landscape of peer-reviewed literature, demonstrating the utility of noninvasive RPM in various patient populations. Future research is needed to determine the effectiveness of RPM via noninvasive digital technologies in delivering patient healthcare benefits and the feasibility of large-scale implementation.*

## Introduction

Remote patient monitoring (RPM) has enhanced clinicians' ability to monitor and manage patients in nontraditional healthcare settings. RPM uses digital technologies to collect health data from individuals in one location, such as a patient's home, and electronically transmit the information to healthcare providers in a different location for assessment and recommendations.^1–3^ More specifically, noninvasive technologies are now commonly being integrated into disease management strategies to provide additional patient information, with the goal of improving healthcare decision-making.^2,4–8^

Digital technologies are continually being adopted as an additional method for healthcare systems to increase patient contact and augment the practice of preventive medicine.^1,2,9^ Healthcare professionals have the ability to share health data with remotely based clinical experts for consultation, saving time and expense for practitioners and patients, and actively managing treatments for those with chronic conditions.^1^ Health data are typically transmitted to healthcare professionals in facilities such as monitoring centers in primary care settings, hospitals and intensive care units, skilled nursing facilities, and centralized management programs, among others.^1,4^ Diversity exists in the design of noninvasive digital technologies for RPM as well as in the role of the patient. For example, some noninvasive digital devices may be automated to capture and transmit health data without any action from the patient (i.e., biosensor or wearable devices); whereas, other technologies may require the patient to submit their own health data through a secure Web site, smartphone, or personal digital assistant (PDA).^1,4^ Common clinical data captured by these technologies include vital signs, weight, blood pressure, oxygen levels, and heart rate.^2,4,10,11^

Publications to date have used different terminology to capture the essence of RPM (e.g., *telehealth*, *telemedicine*, *e-Health*) and the overall language used to describe RPM is inconsistent and still being established.^1–3,9,12,13^ Inconsistency in terminology may be attributed to the evolution of this particular group of technologies. As science and technology continue to advance, RPM has transitioned from capturing remote patient data through telephone interviews and videoconferencing to utilizing automated devices (e.g., biosensor devices) and focusing on particular patient populations (e.g., chronic diseases).^11^ A systematic review of the term *e-health* identified 51 unique definitions for the term with no clear consensus on the meaning.^12^ Another review noted the term *telemedicine* is used interchangeably with the term *e-health* (i.e., communication networks used to deliver healthcare services or health information from one geographical location to another).^11,13,14^ Definitions may also be based on the patient's health condition and how RPM technologies gather and send health data back into the health system.^4,12^ In light of these variances, this study focused on a patient-centered definition of RPM: an ambulatory, noninvasive digital technology used to capture patient data in real time and transmit health information for assessment by a health professional or for self-management.

While many RPM interventions have been adopted on a small scale, large-scale implementation continues to be a challenge. To gain clinical credibility, many RPM technologies are tasked with the “burden of evidence” by publishing in the peer-reviewed literature.^13,15^ Due to the relative infancy of this realm of healthcare, we aim to summarize the current level of evidence to date by completing a comprehensive systematic literature review, and applying the same level of rigor required in the evaluation of other healthcare interventions. Hence, the objective of this systematic review was to identify studies reporting the use of patient-centered RPM via noninvasive digital technologies in the past decade and describe the key trends, including patient and clinical characteristics and health-related findings by these technologies.

## Materials and Methods

A search was conducted in EMBASE (2005–2015) and Ovid MEDLINE (January 1, 2005 to September 15, 2015). The search strategy is provided in Supplementary Table S1 (Supplementary Data are available online at www.liebertpub.com/tmj). An extensive list of search terms was used to identify all appropriate technologies.

The study selection criteria were specified based on the Population, Intervention, Comparator, Outcomes, Timeframe, and Study Design (PICOTS) format.^16^ The patient population included individuals enrolled in a health-related study where the intervention(s) was consistent with our definition of RPM via noninvasive digital technology. We subcategorized interventions based on technology, using the following definitions:

• Any smartphone or PDA device (or associated software/application/text messaging) that is used to transmit patient data to the physician/researcher.
• Any wearable device worn or placed on a body part to record a particular physiological change (e.g., respiratory rate sensors or blood pressure monitors).
• Any biosensor device for recording data from biological or chemical reactions (e.g., pulse oximeters or spirometers).
• A computerized system where data are entered by the patient over an internet connection.
• Multiple components containing more than one technology category above (e.g., biosensor device and computerized system^i^).

The comparators for the review included either standard care or other technologies used to collect patient health data. Outcomes included any health-related outcomes captured by the RPM technology as well as the associated costs (if reported). Study designs included both randomized controlled trials (RCTs), observational studies, and systematic reviews published from January 1, 2005 to September 15, 2015; protocols, nonsystematic reviews, case studies, commentaries, and letters or editorials were excluded.

Specific exclusion criteria were applied to narrow the focus of selected studies to those reporting patient health data captured via noninvasive RPM digital technologies. The predefined exclusion criteria included the following: interventions with invasive or implantable digital technology (e.g., implantable cardiac defibrillators, blood glucose monitors) or nondigital technology (i.e., landline telephone as the only source of data transmission); no remote monitoring of patient data (e.g., treatment algorithm); no real-time data capture (i.e., participants can only access the device at prescheduled times); and if the data acquired by a device were limited to only patient-reported outcomes, survey responses, or drug performance/adherence.

### Study Selection Process

All citations identified from the literature search were screened for relevance. The first level of screening involved an assessment of citation abstracts for relevance by a single reviewer, based on specific exclusion criteria. Two reviewers were consulted to determine whether any uncertain abstracts were to be included. The second level of screening involved a review of the full-text articles identified from the level one screening to determine if these studies met the predefined inclusion and exclusion criteria listed above. This was undertaken by a single reviewer with four additional reviewers consulted to determine whether any uncertain articles were to be included or excluded based on the study selection criteria.

### Quality Appraisal of Literature

The quality appraisal process utilized the Critical Appraisal Skills Programme (CASP) checklists for RCTs, cohort studies, and case–control study designs.^17^ The CASP checklists evaluate the trustworthiness and relevance of studies based on three key areas: study validity (bias), study results (clinical importance and degree of certainty), and relevance (generalizability to patient or population of interest). Quality appraisal was conducted by two independent reviewers for included studies. Instances of disagreement between the two reviewers were identified and evaluated by a third reviewer. Observational, cross-sectional studies are not supported by a CASP quality appraisal checklist. Therefore, the findings from cross-sectional studies should be interpreted with caution.^18^

### Data Collection/Study Variables

Due to the high degree of variability in the study designs and objectives, information was extracted to descriptively assess trends and frequencies of the predefined study variables, including the following: technology category, country setting, patient characteristics, and feedback loop. The health outcomes reported by the included studies were broadly defined as positive, negative, or neutral health outcomes, depending on the impact of the intervention on the study outcomes of interest. Clinical or statistical significance was required for a positive categorization as many studies reported only descriptive findings without any supporting statistical analysis. Cost outcomes were also collected, if reported.

## Results

**Fig. 1.**
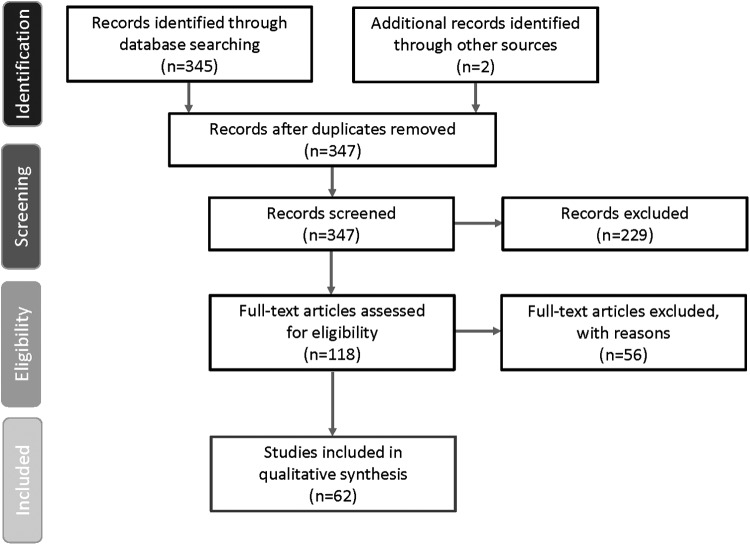
PRISMA diagram of study selection process. PRISMA, Preferred Reporting Items for Systematic Reviews and Meta-Analyses.

The Preferred Reporting Items for Systematic Reviews and Meta-Analyses (PRISMA) flow diagram (*Fig. 1*) graphically represents the citations reviewed, included, or excluded during the course of the systematic review process.^19^ The database search identified 345 articles with two additional articles identified from manual searching. Of the 347 articles identified, 62 articles met the study inclusion criteria. Details of each of these studies can be found in *Table 1*.

**Table 1.** Table of Study Details

FIRST AUTHOR, YEAR	COUNTRY	DISEASE CATEGORY/DISEASE STATE	STUDY DESIGN/STUDY DURATION	SAMPLE SIZE (*N*)	AGE GROUPS (YEARS)	TECHNOLOGY CATEGORY/DEVICE INFORMATION	RESULTS	FEEDBACK LOOP/END USER	FUNDING BODY
Allard, 2014	France	Neurological/Dementia	Cohort/September 2007 to March 2009	60	≥65	Smartphones, PDAs/Mobile assessments of daily life experiences	Positive	Physician	Government
Allen, 2013	United States	Weight management/Obesity	RCT/24 weeks	68	≥20	Smartphones, PDAs/Weight loss application	Positive	Physician and self/Patient	Academic
Anton, 2012	United States	Weight management/Obesity	RCT/104 weeks	671	≥20	Computerized system/Web-based computerized tracking	Positive	Counselors	Government
Barnett, 2011	United States	Substance abuse/Alcohol abuse	Observational/3 weeks	13	≥20	Multiple components/Alcohol monitoring bracelet	Positive	Physician	NR
Bombardini, 2009	Italy	Cardiovascular/Various	Cohort/One session	172	≥20	Wearable device/Accelerometer	Positive	Physician	Academic
Carr, 2013	United States	Weight management/Obesity, overweight, and sedentary	RCT/12 weeks	40	≥20	Multiple components/Activity monitor; pedal machine; motivational Web site; pedometer	Positive	Self/Patient	Academic
Chau, 2012	China	Respiratory/COPD	RCT/8 weeks	40	≥65	Multiple components/A telecare device kit to monitor oxygen saturation, pulse rate, and respiration rate to transmit data to an online network	Neutral	Nurse and Physician	NR
Dansky, 2008	United States	Cardiovascular/Heart failure	RCT/120 days	284	≥65	Multiple components/Telehomecare system through a standard modem, linking a central station to remote units in homes or other settings	Positive	Nurse	Nonprofit foundation
De San Miguel, 2013	Australia	Respiratory/COPD	RCT/24 weeks	71	≥20	Multiple components/HealthHUB (Docobo Ltd., Bookham, Surrey, United Kingdom). Patients measured vital signs and these were transmitted automatically through telephone to a secure Web site	Neutral	Nurse	Government
Dellaca, 2011	Spain	Sleep disorders/Sleep apnea	Observational study/One night	20	40–64	Wearable/Telemetric unit connected to CPAP	Positive	Sleep technician	Government
DeVito Dabbs, 2009	United States	Respiratory/Lung transplant	RCT/8 weeks	30	40–64	Smartphones, PDAs/Health tracking application	Positive	Physician	NR
Dinesen, 2012	Denmark	Respiratory/COPD	RCT/16 weeks	111	≥20	Biosensor/Spirometry	Positive	Physician and Nurse	NR
Dirienzo, 2007	United States	Neurological/Cerebral palsy	Cohort/10 weekly 20-min riding sessions	8	<20	Wearable device/Pulse rate sensor and transmitter	Neutral	NR	NR
Dorsch, 2015	International	Neurological/Stroke rehabilitation	RCT/15 months	125	≥20	Wearable device/Three sets of triaxial accelerometers	Neutral	Physician	Government
Faurholt-Jepsen, 2015 (*Journal of Affective Disorders*)	Denmark	Psychological/Bipolar type 1 or 2	RCT/310 days	33	21–39	Smartphones, PDAs/Android smartphone with the MONARCA system installed and instructed to use the system for daily electronic self-monitoring	Neutral	Self/Patient	Nonprofit foundation
Faurholt-Jepsen, 2015 (*Bipolar Disorders*)	Denmark	Psychological/Bipolar type 1 or 2	RCT/6 months	91	≥20	Smartphones, PDAs/Android smartphone with the MONARCA system installed and instructed to use the system for daily electronic self-monitoring	Neutral	Researcher	Nonprofit foundation
Fox, 2012	Canada	Sleep disorders/Obstructive sleep apnea	RCT/3 months	75	≥20	Wearable device/CPAP device with modem	Positive	Researcher	Industry
Franc, 2014	France	Metabolic disorders/Type 1 diabetes	RCT/6 months	113	≥20	Smartphone, PDAs/Software uploaded into the smartphone	Positive	Medical staff, caregiver	Industry
Fukuoka, 2015	United States	Metabolic disorders/Weight management	RCT/5 months	61	40–64	Smartphones/PDAs/Mobile phone-based program	Positive	Self/Patient	Government and academic
Green, 2008	United States	Cardiovascular/Hypertension	RCT/12 months	730	≥20	Multiple components/Patient Web services, home BP monitoring, and pharmacist-assisted care	Positive	Pharmacist	Government
Greene, 2013	United States	Weight management/Obesity	RCT/6 months	349	≥20	Multiple components/Online social network; accelerometer; scale	Positive	Self/Patient and online social network	Private sector
Guihot, 2007	France	Respiratory/Late-onset noninfectious pulmonary complications	Observational/Median 17 months (4–41) follow-up	37	≥20	Biosensor device/Spirometer	Positive	Physician	NR
Hoefman, 2007	Netherlands	Cardiovascular/Arrhythmia	Observational/30 days	127	≥20	Biosensor/Loop recorder, ECG	Neutral	Cardiologist	NR
Jan, 2007	Taiwan	Respiratory/Allergies, asthma	RCT/12 weeks	164	<20	Multiple components/Electronic peak flow meter and Web site	Positive	Physician	Government
Jehn, 2013	Germany	Respiratory/COPD	RCT/9 months	62	>20	Multiple components/Mobile medical assistant; spirometer; measuring wheel; accelerometer	Positive	Nurse	Government
Jurgens, 2012	Germany	Other/Primary open-angle glaucoma	Observational/6 months	70	≥20	Multiple components/A self-tonometer and BP oscillometric device	Neutral	Ophthalmologist (other)	Government
Kearney, 2009	United Kingdom	Cancer/Chemotherapy-related toxicity	RCT/12–16 weeks	112	40–64	Smartphones/PDAs/ASyMS^©^ mobile phone group completed a symptom questionnaire to measure the incidence, severity, and distress associated with each symptom	Neutral	Physician	Academic
Koff, 2009	United States	Respiratory/COPD	RCT/12 weeks	40	≥20	Multiple components/Health monitoring system; pulse oximeter; FEV1 monitor; pedometer	Positive	A registered respiratory therapist	Academic
Lee, 2013	Korea	Cardiovascular/Acute coronary syndrome	RCT/12 weeks	55	40–64	Wearable device/Wireless monitoring to check heart rates by electrocardiography	Positive	Researcher	Industry and Government
Logan, 2007	Canada	Metabolic disorders/Type 2 diabetes, Hypertension	Cohort/16 weeks	31	≥20	Multiple components/Commercially available Bluetooth-enabled BP monitoring device	Positive	Physician	Government
Logan, 2012	Canada	Metabolic disorders/Type 2 diabetes	RCT/1 year	110	>20	Multiple components/Commercially available Bluetooth-enabled BP monitoring device	Positive	Physician	Nonprofit foundation
Luley, 2014	Germany	Metabolic disorders/Type 2 diabetes, Obesity, Weight management	RCT/6 months	70	40–64	Multiple components/Health monitoring system, including weighing scales and accelerometer	Positive	Self/Patient	NR
Mehring, 2013	Germany	Weight management/Obesity	RCT/12 weeks	186	40–64	Computerized system/Web site program for individual coaching	Positive	Physician or Nurse	Private Sector
Mendelson, 2014	France	Sleep disorders/Obstructive sleep apnea	RCT/16 weeks	107	≥20	Multiple components/CPAP and smartphone to transmit clinical information	Positive	Physician	Private Sector
Minassian, 2010	United States	Psychological/Bipolar type 1 or 2, schizophrenia	Observational/Three 5-min epochs	28	21–39	Wearable device/Upper body garment ECG	Neutral	NR	Government; Nonprofit organization
Nollen, 2014	United States	Weight management/Obesity	RCT/12 weeks	51	<20	Smartphones, PDAs/Handheld computer program	Neutral	Self/Patient	Government
Olson, 2007	United States	Cardiovascular/Palpitations, presyncope, syncope	Observational/July 2003 to August 2004	122	40–64	Biosensors/Sensor records two ECG channels with a separate monitoring device	Positive	Physician	NR
Palmas, 2008	United States	Metabolic disorders/Type 2 diabetes	Cohort/mean follow-up time 32.1 ± 8.4 months	392	≥65	Wearable devices/Oscillometric device	Neutral	Physician	Government
Pedone, 2015	Italy	Cardiovascular/Heart failure	RCT/6 months	96	≥65	Multiple components/Sphygmomanometer, a scale: pulse oximeter	Positive	Physician	NR
Pedone, 2013	Italy	Respiratory/COPD	RCT/9 months	99	≥65	Multiple components/Telemonitoring system: wearable device, pulse-oximeter, cellular telephone	Positive	Physician	Government
Piga, 2014	Italy	Other/Systemic sclerosis and rheumatoid arthritis	RCT/12 weeks	40	40–64	Biosensor/Portable briefcase includes sensor tools	Positive	Physician	Government
Rasmussen, 2005	Denmark	Respiratory/Asthma	RCT/6 months	300	≥20	Computerized system/Internet-based asthma management tool	Positive	Physician	Academic, Industry, and Private
Rogers, 2014	United States	Psychological/Trichotillomania	RCT/10 weeks	60	≥20	Computerized system/Patient Web site	Positive	Self/Patient	Government
Ryan, 2012	United Kingdom	Respiratory/Asthma	RCT/6 months	288	≥20	Smartphones/PDAs/Asthma phone application	Neutral	Nurse	Nonprofit organization
Salarian, 2007	Switzerland	Neurological/Parkinson's disease	Case-control/∼4–5 min	20	≥20	Wearable device/Biaxial accelerometer, gyroscope, data logger	Positive	Not defined	Nonprofit foundation
Scalvini, 2005	Italy	Cardiovascular/Palpitations	RCT/7 days (or two recordings)	310	40–64	Multiple components/One-lead ECG trace sent it to telemedicine center	Positive	Nurse or cardiologist	NR
Shane-McWhorter, 2014	United States	Metabolic disorders/Type 2 diabetes, hypertension	Observational/Average duration 7 months	109	40–64	Multiple components/One of two telemonitoring delivery methods: touch-screen or an IVR system both included BP monitor heart rate monitoring and patients were provided a digital scale	Positive	Pharmacist, Healthcare educators	Government
Shany, 2010	Australia	Respiratory/COPD	RCT/12 months	80	≥20	Multiple components/A home monitor comprising a fixed portal, which was used to record daily physiological parameters as well as enter weight, body temperature, and blood glucose levels (if diabetic)	Neutral	Nurse	NR
Spring, 2013	United States	Weight management/Obesity	RCT/12 months	69	≥20	Smartphones/PDAs/PDA to record food intake, weight, and physical activity	Positive	A behavioral coach (Counselor)	Government
Stone, 2010	United States	Metabolic disorders/Type 2 diabetes	RCT/3 months	150	≥20	Biosensor/Home telemonitoring device with monitoring of blood glucose, blood pressure, and weight	Positive	Nurse	Government
Su, 2014	Taiwan	Neurological/Parkinson's disease	Case series/One session	5	≥65	Wearable device/Triaxial accelerometer	Neutral	Researchers	Nonprofit organization
Tabak, 2012	Netherlands	Respiratory/COPD	Cohort/4 days	39	≥20	Multiple components/Activity sensor and smartphone were worn on the subject's belt	Neutral	Not defined	NR
Thomas, 2015	United States	Weight management/Obesity	RCT/6 months	154	≥20	Computerized system/Weight-loss Web site	Positive	Physician	Government
van der Meer, 2009	Netherlands	Respiratory/Asthma	RCT/12	200	≥20	Multiple components/Spirometer and Web site	Positive	Asthma nurse	Nonprofit organization
Wakefield, 2011	United States	Metabolic disorders/Type 2 diabetes, Hypertension	RCT/52 weeks	302	≥20	Multiple components/The home telehealth device to enable data transmission	Positive	Nurse	Government
Wakefield, 2012	United States	Metabolic disorders/Type 2 diabetes, hypertension	RCT/52 weeks	302	≥20	Multiple components/The home telehealth device to enable data transmission	Positive	Nurse	Government
Wang, 2012	United States	Weight management/Obesity	RCT/12 months	210	≥20	Smartphones/PDAs/PDA recorded food entry and physical activity	Positive	Self/Patient	Government
Webber, 2013	United States	Weight management/Obesity	RCT/12 weeks	50	≥20	Computerized system/Online self-monitoring	Positive	Self/Patient	Nonprofit organization and private sector
Widmer, 2015	United States	Cardiovascular/Cardiovascular rehabilitation	Cohort/3 months	42	≥20	Multiple components/Personal health assistant through online and smartphone-based platforms	Positive	Self/Patient	Nonprofit foundation
Wijsman, 2013	Netherlands	Weight management	RCT/13 weeks	235	≥20	Multiple components/Accelerometer-based activity monitor, a personal Web site, and a personal e-coach	Positive	Self/Patient and e-coach (counselor)	Nonprofit organization and private sector
Woodend, 2008	Canada	Cardiovascular/Heart failure and Angina	RCT/12 weeks	249	≥20 y	Biosensor/Transmission of ECG	Positive	Nurse	Nonprofit foundation and industry.
Zheng, 2014	China	Cardiovascular/Healthy control subjects	Case series/24 h	10	21–39	Wearable device/Wearable ECG: two e-textile electrodes were sewed in the armband and one placed on the right side of the thorax	Positive	Researcher	Government

COPD, chronic obstructive pulmonary disease; CPAP, continuous positive airway pressure; ECG, electrocardiogram; FEV, forced expiratory volume; IVR, interactive voice response; NR, not reported; PDA, personal digital assistant; RCT, randomized controlled trial.

### Quality Appraisal

The majority of the 62 studies included in the systematic review used an RCT design. Most of the RCT studies (*N* = 44) received positive responses to the CASP checklist criteria, although one of the criteria regarding blinding of patients and study personnel (to the RPM intervention) was reported by only 10 of the RCTs. The cohort studies (*n* = 7) and case–control study (*n* = 1) also had primarily positive responses after being reviewed by the CASP checklists, whereas the remaining studies (*n* = 10) were observational, cross-sectional study designs with no quality appraisal performed.

### Overall Trends: RPM Noninvasive Digital Technology

**Fig. 2.**
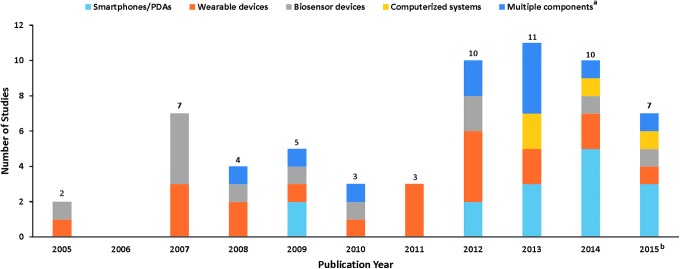
Identified RPM via Noninvasive Digital Technology Studies (January 1, 2005 to September 15, 2015) ^a^Multiple components refer to studies containing more than one technology category. ^b^Searches ended September 15, 2015. PDAs, personal digital assistants; RPM, remote patient monitoring.

RPM with noninvasive digital technologies is becoming more available for monitoring and collecting patient healthcare information. *Figure 2* illustrates the number of published RPM with noninvasive digital technology studies, by technology category, included in the systematic review over the last decade. Funding of included studies were mainly from government and nonprofit organizations.

**Fig. 3.**
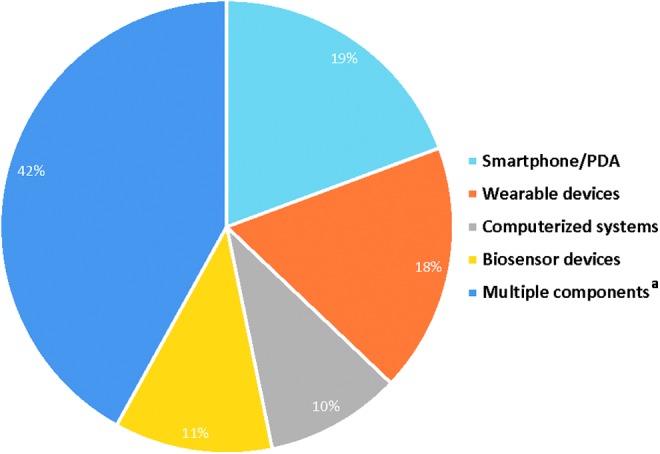
RPM through Noninvasive Digital Technologies ^a^Multiple components refer to studies containing more than one technology category.

The breakdown of technology categories included in the current systematic review is illustrated in *Figure 3*. Overall, 12 studies were identified using a smartphone or PDA,^20–31^ 11 studies utilized wearable devices,^32–42^ 7 studies utilized biosensor devices,^43–49^ 6 studies included computerized systems,^50–55^ and 26 studies comprised multiple technologies.^ii,56–81^

The patient populations of the included studies were predominantly adults, 20 years of age or older, with only 5% of studies including children or adolescents (<20 years of age). Many studies recruited specific adult age groups: young adults between 21 and 39 years of age (5% of studies), older adults between 40 and 64 years of age (18%), and seniors ≥65 years old (10%). Overall, the majority (61%) of studies included a mix of younger and older adult populations. The clinical characteristics of the included studies showed a dominance of chronic disease populations. The most prevalent conditions included the following: 23% respiratory diseases (e.g., asthma), 18% weight management, 18% metabolic diseases (e.g., type 1 and 2 diabetes), and 16% cardiovascular diseases (e.g., heart failure.)

All studies included elements of a feedback loop, however, the end user or recipient of the captured data varied greatly across the included studies. The majority included physicians or nurses as the main recipient of patient health data (31% and 16%, respectively). In 18% of studies, the end user of the captured data was the patient. These studies primarily utilized smartphones/PDAs or computerized systems to enable self-monitoring of patient lifestyle behaviors (i.e., caloric intake and exercise).

### Key Trends by Noninvasive Digital Technology

The key trends for each of the technology categories are listed in *Table 2*, diversity among the technologies was shown across all of the study variables. Only studies on computerized systems were found to be primarily conducted within the United States, and studies on smartphones/PDAs were evenly split between U.S. and non-U.S. settings. In terms of clinical and patient characteristics, patients with respiratory disorders/diseases and metabolic conditions were primarily monitored through multicomponent interventions. In contrast, weight management was mainly monitored through computerized systems, and patients with cardiovascular diseases were mainly monitored using biosensor devices.

**Table 2.** Key Trends by Noninvasive Digital Technology (Number of Studies)

	SMARTPHONES/PDAS	WEARABLE DEVICES	BIOSENSOR DEVICES	COMPUTERIZED SYSTEM	MULTIPLE COMPONENTS
Number of studies, *N* = 62, *n* (%)	12 (19)	11 (18)	7 (11)	6 (10)	26 (42)
Country, *n* (%)					
Non-U.S.^a^	6 (50)	7 (64)	5 (71)	2 (33)	16 (62)
U.S.	6 (50)	4 (36)	2 (29)	4 (67)	10 (38)
Disease category, *n* (%)					
Cancer	1 (8)	—	—	—	—
Cardiovascular	—	3 (27)	3 (43)	—	5 (19)
Metabolic disorders	2 (17)	1 (9)	1 (14)	—	6 (23)
Neurological	1 (8)	4 (36)	—	—	—
Psychological	2 (17)	1 (9)	—	1 (17)	—
Respiratory	2 (17)	—	2 (29)	1 (17)	9 (35)
Sleep disorders	—	2 (18)	—	—	1 (4)
Substance abuse	—	—	—	—	1 (4)
Weight management	4 (33)	—	—	4 (67)	3 (12)
Other/Multiple^b^	—	—	1 (14)	—	1 (4)
Age category, *n* (%)					
<20 years old	1 (8)	1 (9)	—	—	1 (4)
21–39 years old	1 (8)	2 (18)	—	—	—
40–64 years old	3 (25)	2 (18)	2 (29)	1 (17)	3 (12)
≥65 years old	1 (8)	1 (9)	—	—	4 (15)
>20 years old^c^	6 (50)	4 (36)	5 (71)	5 (83)	18 (69)
Not reported		1 (9)			
Feedback loop, *n* (%)					
Counselors	1 (8)	—	—	1 (17)	—
Nurse	1 (8)	—	2 (29)	—	7 (27)
Pharmacist	—	—	—	—	1 (4)
Physician	3 (25)	3 (27)	4 (57)	2 (33)	7 (27)
Researchers	1 (8)	4 (36)	—	—	—
Self/Patient	4 (33)	—	—	2 (33)	5 (19)
Not reported	—	3 (27)	—	—	1 (4)
Other^d^	2 (17)	1 (9)	1 (14)	1 (17)	5 (19)
Results, *n* (%)					
Negative	—	1 (9)	—	—	—
Neutral	5 (42)	5 (45)	1 (14)	—	5 (19)
Positive	7 (58)	5 (45)	6 (86)	6 (100)	21 (81)

^a^ Non-U.S. countries included Canada, China, Taiwan, Korea, and several European countries (France, Italy, Spain, Denmark, Netherlands, Germany, Switzerland, and the United Kingdom).

^b^ Disease category: “Other/multiple” includes patients with leg ulcers, glaucoma, systemic sclerosis, and rheumatoid arthritis.

^c^ Age category: “Adults (>20 years)” refers to studies with more than one adult age category included (e.g., 35–70 years old).

^d^ Feedback loop: “Other” refers to multiple categories of feedback loop categories (i.e., physician and researcher), other healthcare professionals not listed, caregivers, or studies which did not explicitly report the end-user of the data.

The vast majority of studies including adults ≥20 years (incorporating both younger and older adult populations), primarily utilizing multiple-component technologies. Of the three studies including child/adolescent populations, multicomponent interventions, smartphones/PDAs, or wearable devices were used. Similarly, the three studies that specifically recruited young adults (20–39 years) employed smartphones/PDAs or wearable devices. In terms of the feedback loop, smartphones/PDAs and computerized systems most often used the patient as the primary recipient of health data. Wearable devices most often transmitted data to study researchers; whereas biosensor devices and multicomponent interventions predominantly transmitted data to physicians or nurses. Although most of the studies included were RCT designs, the health outcomes reported were diverse and mainly descriptive in nature; therefore, outcomes were classified as primarily positive, negative, or neutral to understand the overall trends. Overall, most studies reported positive health outcomes,^iii^ including the computerized technologies (100%), followed by biosensor devices (86%), multicomponent interventions (81%), and smartphones/PDAs (58%). Only studies on wearables devices found an even split between positive and neutral outcomes.^iv^ Of the six studies that reported cost associated with utilizing an RPM technology, these were also neutral or positive compared with the control group (data not shown).^29,43,60,66,69,81^

## Discussion

RPM is an evolving and growing area of healthcare innovation in terms of research potential and improvement in healthcare services and delivery.^5–8,82^ In the past, *telemedicine* and *e-health* were common terms used more broadly to define patient data captured from landlines or videoconferencing interventions. Over the last 10 years, RPM via noninvasive digital technologies has rapidly become a more common way to obtain patient health data. Our systematic literature review is unique in that we identified studies utilizing RPM via noninvasive technologies to investigate the application of these technologies among diverse patient populations and various health conditions.

The majority of studies included in this systematic review were RCTs of multicomponent technologies in adult populations. Chronic conditions, including respiratory diseases, weight management, metabolic diseases, and cardiovascular diseases, were the focus of noninvasive digital RPM interventions, which indicates the high burden of chronic disease to the health system. Healthcare settings where a physician or nurse collected and provided feedback to the patient regarding the remotely captured data represented the majority of studies; the data feedback loop is a critical component in ensuring that RPM interventions have a high impact on patient health. Due to the descriptive nature of many of the included studies, health outcomes were categorized as positive, negative, or neutral (rather than individual measures of efficacy or effectiveness) to allow for an assessment of the overall trends in improving outcomes across all the technologies. The health outcomes data reported were classified as mostly positive across all of the included studies. Key outcomes included self-management (i.e., taking blood pressure or tracking weight) shown to help symptoms severity and reduce patient visits and/or hospitalizations,^59,67,79^ as well as quality of life improvement through activation of self-care behaviors.^22,66^

A limited number of studies have reported healthcare data on the use of RPM via noninvasive digital technologies in the peer-reviewed literature.^83,84^ Large-scale implementation is challenging in terms of budget (cost of technology and infrastructure to monitor), educating patients in the use of devices, training providers in the collection and interpretation of results, as well as incorporation of the remote patient data into routine clinical practice.^6,84,85^ To our knowledge, this study is the first systematic review of RPM using noninvasive digital technologies examining any disease or condition spanning the last decade in the peer-reviewed literature. Similar to systematic reviews of other clinical interventions, we purposely applied the same level of methodological rigor, including quality appraisal, to identify the best available evidence to describe the key trends, patient and clinical characteristics, and outcomes measured by these technologies.

Several studies identified gaps that may be addressed by future research, including patient activation and costs. In terms of patient activation, new technologies such as smartphones/PDAs not only reduce resources in terms of patient visits but they may also promote health-related behavioral changes in patients by delivering convenient and individually tailored interventions.^5–8,21,86^ Furthermore, wireless transmission of patient data directly from devices to a centralized system may be the most cutting-edge technology available.^5^ These devices (typically biosensors or wearables) reduce patient burden and human error associated with manual entry and data transmission from the peripheral devices to the centralized data system.

In this systematic review, the few studies that reported cost outcomes found many costs associated with utilizing an RPM technology to be neutral or cost-saving compared with the control group.^29,43,60,66,69,81^ The majority of patients in studies reporting cost were >50 years of age, which may be due to the value of RPM data being highest among seniors due to the demand on provider's time.^15,87^ The growing interest in RPM for programs such as Medicare is expected to increase as the senior U.S. population grows and future studies may begin to explore cost-savings associated with these technologies.^88^ However, a recent RCT study found little evidence of differences in short-term healthcare utilization and costs between patients with chronic conditions monitored through mobile health or digital medicine technology versus standard disease management.^89^ To better understand the appropriate use of RPM interventions using noninvasive digital technologies, future research should explore long-term healthcare utilization and costs in various patient populations such as those with chronic diseases.

## Limitations

There are a few limitations that should be noted for our systematic review. The search string was very specific and may not have captured all articles. Although the majority of studies were RCT designs, observational cross-sectional studies were included, but no quality appraisal was performed. These studies are unlikely to impact the overall findings due to the descriptive nature of the results. In addition, the majority of studies were exploratory or pilot designs, with a high degree of variability in the objectives, populations, and outcomes reported; therefore, the information extracted was limited to broad categorization, subject to prespecified definitions, to assess commonality and trends. Many of the studies included in the systematic review enrolled relatively small patient populations; future clinical trials should assess the sample size required to have an adequately powered design and allow for a more generalizable population to assess the effectiveness of RPM technology on the outcomes of interest. In addition, the majority of the included studies reported short trial durations; however, to accurately assess the effectiveness of these technologies, the duration should align with the study objectives and clinical outcomes assessed (e.g., readmission rates). Discretion was used with the “feedback loop/end user” variable as this was not clearly described in many of the articles; however, we still felt it was an important aspect of these interventions and highly relevant to their overall value in healthcare. Many of the studies included only descriptive results; lack of robust measurement on the benefit of RPM on patient outcomes and care delivery was demonstrated. In terms of implementation, most studies did not report whether the data was ultimately added to the health system records, as previously stated this may be due to the exploratory nature of these studies.

## Conclusions

Despite its potential, the rapid development of technological capabilities in the healthcare field has outpaced the capacity to implement many novel RPM interventions into real-world practice. Hence, scant evidence demonstrating improved health outcomes with noninvasive RPM interventions is available and even fewer studies have demonstrated any cost benefit. Based on this systematic review, there is a key trend toward using multicomponent interventions for the monitoring of chronic conditions in older populations. Further research utilizing robust study designs is needed to assess the efficacy and value of RPM technology for decision makers, developers, researchers, clinicians, and investors. This study will help decision makers better understand the current state of evidence available in peer-reviewed literature and assist in the planning of future studies that could address the existing gaps identified in this systematic review. Large-scale implementation is required to confirm the benefits of RPM across sectors and populations in the health systems and identify the “optimal” patient for maximum utility of RPM. Barriers to implementation should also be assessed, including provider training, data reliability, security, and incorporation of RPM data into routine care.

## Supplementary Material

Supplemental data

## Notes

^i^ For example: Greene et al.^62^ incorporated data captured from an online social network, accelerometer, and a scale.

^ii^ For example: Greene et al.^62^ incorporated data captured from an online social network, accelerometer and a scale.

^iii^ Positive outcomes example: Allard et al.^20^ reported that imaging markers of dementia were significantly associated with mobile assessments of semantic memory performance.

^iv^ Neutral outcomes example: De San Miguel et al.^60^ reported the telehealth group had nonsignificant reductions in emergency department visits, hospital admissions, and length of stay in comparison with the control group.
