# Supplementary material for: Remote Patient Monitoring via Non-Invasive Digital Technologies: A Systematic Review
Source: Telemed J E Health. 2017 Jan 1;23(1):3–17. doi: 10.1089/tmj.2016.0051 (PMC5240011; doi:10.1089/tmj.2016.0051)
Supplement: Supplemental data [file Supp_Table1.pdf]

Supplementary Data

Supplementary Table S1. Search Strategy: EMBASE (<1996 to 2015) and Ovid MEDLINE (<1946 to September Week 1 2015>)

SEARCH NUMBER	SEARCH TERM	RESULTS
1	remote monitoring.mp. or remote sensing/	5727
2	self-monitoring/	4932
3	self-tracking.mp.	36
4	remote tracking.mp.	13
5	home monitoring/	2693
6	wireless monitoring.mp.	254
7	online monitoring/	1416
8	online tracking.mp.	66
9	telemonitoring/	1374
10	ambulatory monitoring/	13898
11	1 or 2 or 3 or 4 or 5 or 6 or 7 or 8 or 9 or 10	29169
12	observational study/	91448
13	controlled study/	4115502
14	12 or 13	4186215
15	(e-health or m-health or telehealth or telemedicine or digital or mobile or social network or smartphone or mobile phone or cell phone or wearable or Web based or internet based).mp. [mp=ti, ab, hw, tn, ot, dm, mf, dv, kw, nm, kf, px, rx, ui]	402914
16	11 and 14 and 15	501
17	limit 16 to (human and english language and yr="2005-Current")	392
18	implantable cardioverter defibrillator/	39827
19	17 not 18	374
20	implant$.mp.	731239
21	19 not 20	357
22	artificial heart pacemaker/	14785
23	21 not 22	355
24	analog digital converter/	816
25	23 not 24	353
26	echocardiography/	186792
27	25 not 26	350
28	remove duplicates from 27	345
